# Panax Notoginseng Saponins as a Novel Nature Stabilizer for Poorly Soluble Drug Nanocrystals: A Case Study with Baicalein

**DOI:** 10.3390/molecules21091149

**Published:** 2016-08-30

**Authors:** Yuanbiao Xie, Yueqin Ma, Junnan Xu, Yang Liu, Pengfei Yue, Qin Zheng, Pengyi Hu, Ming Yang

**Affiliations:** 1 Key Laboratory of Modern Preparation of TCM, Ministry of Education, Jiangxi University of Traditional Chinese Medicine, Nanchang 330004, China; yuanbiaoxie@163.com (Y.X.); JunnanXuJX@126.com (J.X.); kjwxjs2013_2@126.com (Y.L.); jxtcmi412@126.com (Q.Z.); shuotcm@126.com (P.H.); jxtcmi215@126.com (M.Y.); 2 Department of Pharmaceutics, 94th Hospital of People’s Liberation Army, Nanchang 330000, China; Yueqinma@126.com

**Keywords:** nanocrystals, nanosuspensions, panax notoginseng saponins, redispersibility, freeze-drying, spray-drying

## Abstract

This study is aimed at seeking a nature saponin-based stabilizer for drug nanosuspensions. A poorly soluble drug (baicalein, BCL) was used as a model drug. BCL nanosuspensions with particle size of 156 nm were prepared by means of homogenization and converted into BCL nanocrystals (BCL-NC) stabilized with panax notoginseng saponins (PNS). It was found that PNS was able to prevent the aggregation of BCL-NS during storage and improve the redispersibility of BCL-NC after freeze-drying and spray-drying, compared with polymer stabilizer PVPK30. The freeze-dried and spray-dried BCL-NC with PNS exhibited excellent performance as evidenced by scanning_electron_microscope (SEM) analysis. It was the reason that PNS possessed the interfacial property (41.69 ± 0.32 mN/m) and electrostatic effect (−40.1 ± 1.6 mV), which could easily adsorb onto the surface of hydrophobic BCL nanocrystals and prevent from its aggregation. It is concluded that PNS can be used as an effective nature stabilizer for production of drug nanocrystals.

## 1. Introduction

Nanocrystals suspensions (also named as nanosuspensions) is colloidal dispersion system for poorly soluble drugs generally produced in liquid media, which possess numerous excellent advantages that enhance the solubility and dissolution velocity of poorly soluble drugs due to their small particle size and high surface area [1,2], and strengthen the adhesion to biological membrane, and prolong adhesion time and detention time [3,4]. However, nanocrystals at suspension state are essentially thermodynamically unstable, which would result in flocculation, aggregation or crystal growth due to Ostwald ripening [5]. In terms of the stability as well as the convenience for the patient, drug nanosuspensions are usually converted into dried nanocrystals powders by spray-drying and freezing-drying [6,7,8]. However, this drying process can generate a series of stresses (due to freezing strength for freeze-drying or thermal intensity for spray-drying) and induced irreversible agglomeration of individual nanocrystals particles, which can prevent nanocrystals from recovering to original nanosuspensions states followed by rehydration (redispersibility) [9]. Therefore, proper selection of stabilizers is required during the preparation of nanocrystals. The most common approaches of stabilization are steric and/or electrostatic technique. Steric stabilization effect is achieved by adsorbing polymers stabilizer onto the drug particle surface, whereas electrostatic stabilization is obtained by adsorbing charged molecules, both ionic surfactants and charged polymers, onto the particle surface [10]. Common pharmaceutical excipients that are suitable for use as polymeric stabilizers include hydroxypropyl methylcellulose (HPMC), hydroxypropylcellulose (HPC), as well as polyvinyl pyrrolidone (PVPK30). Non-ionic surfactant stabilizers, such as polysorbate (Tween 80) and anionic surfactants such as sodium dodecyl sulfate (SDS) can also be used [11,12]. However, polymers stabilizers may be not beneficial to homogenization process especially for hydrophobic drugs [13]. Further, surfactant stabilizers such as sodium dodecyl sulfate (SDS) at high concentration, sometimes offer challenge in producing patient friendly dosage form due to local gastric irritation. Therefore, application of friendly stabilizer is crucial for stability of nanosuspensions during nanosization and solidification.

**Figure 1:**
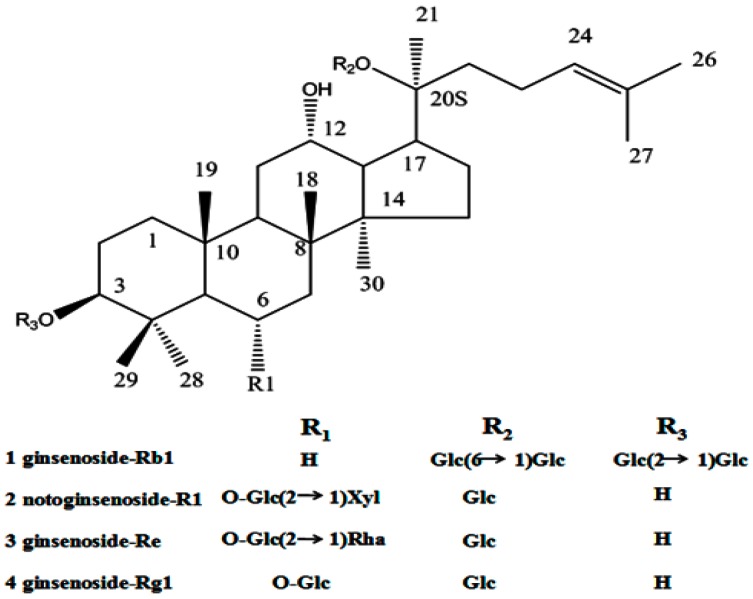
Structure of dammarane saponins **1**–**4** from panax notoginseng saponins.

Saponins as a kind of natural stabilizer, widely distributed in the plant kingdom, include a diverse group of compounds characterized by their structure containing a steroidal or triterpenoid aglycone and one or more sugar chains. Saponins have been widely used in food and other industrial applications mainly as surface active and foaming agents [14]. A natural saponin-based surfactant has been increasing interest within the food industry in replacing synthetic ingredients with natural “label friendly” alternatives [15,16]. Therefore, saponin has also been used as natural emulsifiers that can be successfully used in emulsion-based food and beverage products [17]. Panax notoginseng (sanqi or tienchi in Chinese), is the roots of the *Panax notoginseng* (Burk) F.H. Chen. In China, panax notoginseng has been widely accepted in food and therapy. Panax notoginseng saponins (PNS) extracted from panax notoginseng consist of various saponins (Figure 1), such as ginsenoside Rb1, ginsenoside Rg1, ginsenoside Re and notoginsenoside R1. Therefore, panax notoginseng saponins are firstly proposed as an alternative stabilizer for drug nanocrystals, and its protective effect during conversion of drug nanocrystals is studied.

The model drug was baicalein (BCL), which had variety of pharmacological effects [18], such as anti-cancer [19], anti-HIV [20], anti-inflammatory [21], anti-bacterial [22] and anti-adipogenic [23] activities. Unfortunately, BCL is poorly water soluble and highly permeable, which has very poor oral absorption and low bioavailability [24]. The objectives of this study were as follows: (1) BCL nanocrystals suspension (BCL-NS) stabilized by PNS were prepared by homogenization technology; (2) BCL-NS/PNS was processed into the dried nanocrystals powders (BCL-NC); and (3) the influence of the different solidification stresses (freeze, freeze-drying and spray-drying) on the redispersibility of BCL-NC was investigated. The performance of PNS was compared to those of synthetic stabilizers that are currently widely used in production of nanocrystals.

## 2. Results and Discussion

### 2.1. Influences of PNS on the Dispersion Efficiency and Stability of BCL-NS during Homogenization

**Figure 2:**
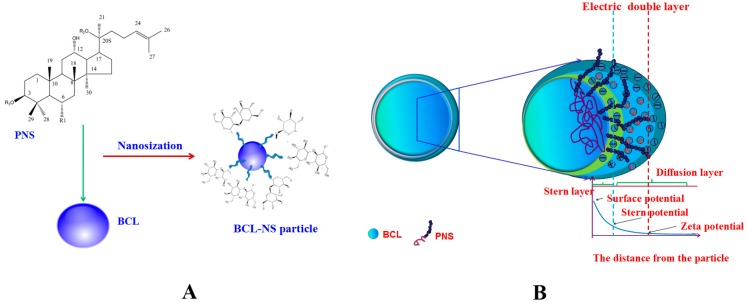
The schematic image of BCL-NS/PNS (**A**) and stabilization mechanism of BCL-NS/PNS (**B**).

BCL-NS was prepared by high pressure homogenization. The dispersion efficiency of PNS, PVPK30, Tween 80 and HPMC on BCL-NS was investigated. Suspensions were generally classified as nanosuspensions when the average 50% volume percentiles (D_50_) values were below 1 µm [13]. The average D_50_, span values and stability index (SI) for different BCL-NS are listed in Table 1. The mean particle size of the freshly prepared BCL-NS/PNS, BCL-NS/PVPK30, BCL-NS/Tween 80 and BCL-NS/HPMC was on the range 140–160 nm. These results showed that the BCL-NS was successfully prepared in terms of stabilizer PNS, PVPK30, Tween 80 and HPMC. The viscosity and surface tension results of different stabilizers solutions (see Table 2) indicated that the viscosity of PNS solution was smaller than those of polymers HPMC and PVPK30 solutions at equivalent concentration, while the surface tension of PNS solution was similar with those of surfactant Tween 80 solution. Therefore, the surface activity of PNS might play an important role on the nanodispersion of BCL-NS during homogenization process. It might be the reason that PNS could absorb onto the surface of BCL-NC and prevent from aggregation of BCL-NC during homogenization and storage process (Figure 2A).

The results of short stability study (Table 1) demonstrated that the BCL-NS/PNS (0.969 ± 0.012) and BCL-NS/PVPK30 (1.021 ± 0.006) were relatively stable at storage for one month, compared with BCL-NS/Tween 80 (1.056 ± 0.004) and BCL-NS/HPMC (1.443 ± 0.005). The Zeta potential (ZP) of BCL-NS/PNS, BCL-NS/PVPK30, BCL-NS/Tween 80, and BCL-NS/HPMC was −40.1 mV ± 1.6 mV, −31.7 mV ± 2.3 mV, 33.4 mV ± 1.4 mV and −29.1 mV ± 3.1 mV, respectively. It indicated that the BCL-NS/PNS possessed lower surface potential, compared with BCL-NS/PVPK30, BCL-NS/Tween 80, and BCL-NS/HPMC (Table 1). As depicted in Figure 2B, PNS could prevent particle-particle interactions and the subsequent agglomeration of BCL-NS during storage process, due to the electrostatic repulsion of stabilizers adsorbed onto surface of nanocrystals [25,26].

### 2.2. Effect of PNS with or without Other Cryoprotectants on Redispersibility of BCL-NC during Freeze-Drying Process

Freezing is well known to be a crucial process of freeze-drying [9]. During freezing of liquid nanocrystals, absence of myriad stabilizers can induce formation of non-frozen liquid nanocrystals phase resulting in phase separation. This might cause separation of BCL-NC in the non-frozen liquid phase increasing the probability of particulate aggregation. In order to prevent irreversible aggregation and preserve the redispersibility of BCL-NC, the freezing step is more important than the subsequent sublimation step. Hence, before sublimation step, the influence of PNS combined with cryoprotectants on the redispersibility of BCL-NS at different freezing temperature stresses was investigated. Because of its excellent performance, the influence of PVPK30 on the redispersibility of BCL-NS was also investigated and used as a reference.

**Figure 3:**
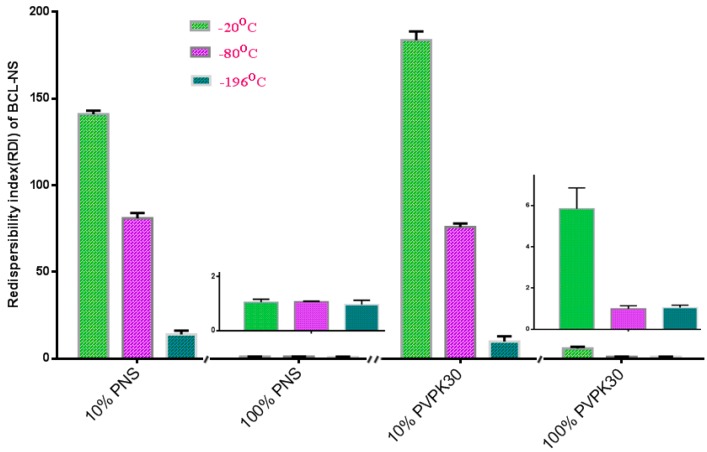
Redispersibility of the frozen BCL-NS/PNS and BCL-NS/PVPK30 at −20 °C, −80 °C and −196 °C (mean ± S.D., *n* = 3).

Figure 3 shows the redispersibility of the frozen BCL-NS stabilized by PNS or PVPK30 at −20 °C, −80 °C and −196 °C. Redispersibility (RDI) of BCL-NS with 10% concentration (*w/w*) of PNS (BCL-NS/10% PNS) at −20 °C, −80 °C and −196 °C was 140.96 ± 2.07, 80.95 ± 3.74 and 14.01 ± 3.41, respectively. Furthermore, redispersibility of BCL-NS with 10% concentration (*w/w*) of PVPK30 (BCL-NS/10% PVPK30) was similar with that of BCL-NS/10% PNS. These demonstrated that the freezing stress could result in aggregation of BCL-NS. The extent of aggregation of BCL-NS frozen at −20 °C was much worse compared to −80 °C and −196 °C. It seemed that the aggressive freeze temperature could provide favorable condition that prevent from aggregation of BCL nanocrystals during freezing process, which was consistent with previous reports [9,27]. It was also concluded that 10% concentration of PVPK30 or PNS could not prevent the aggregation of BCL-NS. However, 100% concentration of PNS could improve redispersibility of the frozen BCL-NS at different freezing stress. 

**Figure 4:**
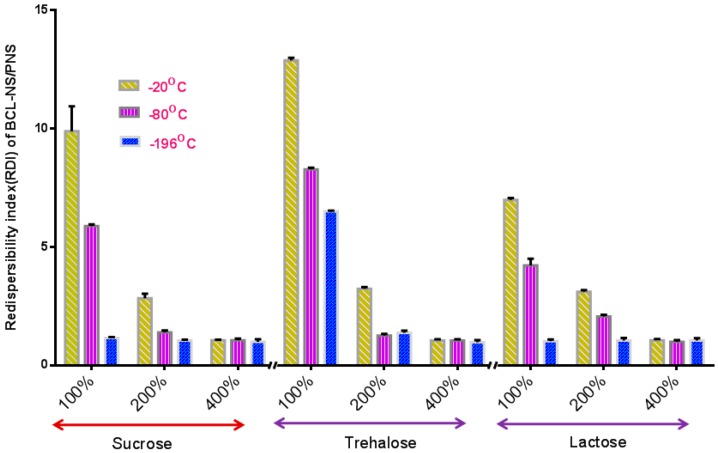
Redispersibility of BCL-NS/10% PNS combined with different concentrations of cryoprotectants during freezing (mean ± S.D., *n* = 3).

**Figure 5:**
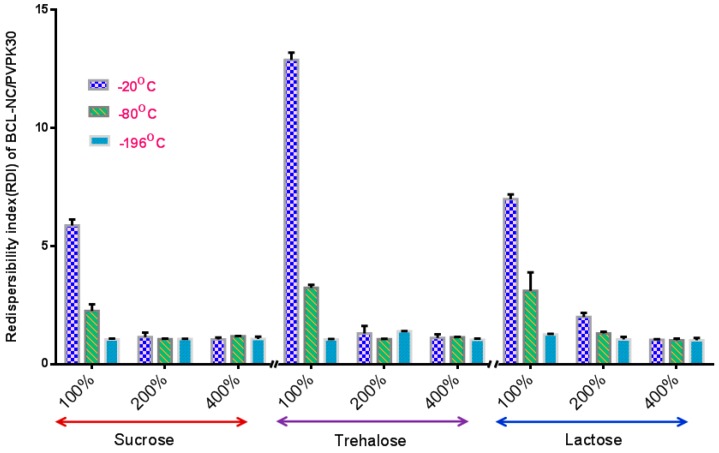
Redispersibility of BCL-NS/10% PNS combined with different concentrations of cryoprotectants during freezing (mean ± S.D., *n* = 3).

The stabilization effect of 10% concentration of PNS combined with cryoprotectants (sucrose, trehalose and lactose) for BCL-NS during freeze-drying was investigated, and stabilizer PVPK30 was also used as reference. Figure 4 and Figure 5 show the redispersibility of BCL-NS/10% PNS and BCL-NS/PVPK30 combined with different concentrations of cryoprotectants during freezing. The result illustrated that the 10% concentration of PNS combined with 400% concentration of cryoprotectants (sucrose, trehalose and lactose) possessed excellent performance for BCL-NS during freezing, compared to PVPK30. Furthermore, redispersibility (RDI 1.12 ± 0.09) of BCL-NS/10% PNS with 200% trehalose was also acceptable, compared to PVPK30 (RDI 1.37 ± 0.11) of BCL-NS/10% PVPK30.

**Figure 6:**
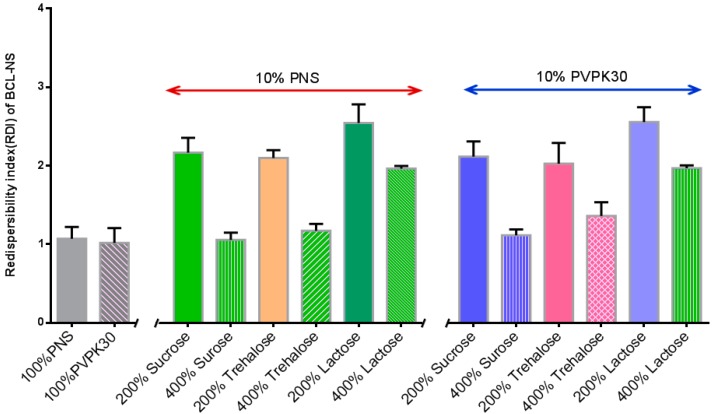
Redispersibility of the freeze-dried BCL-NC with 10% PNS and combined with different concentrations of matrix formers (mean ± S.D., *n* = 3).

**Figure 7:**
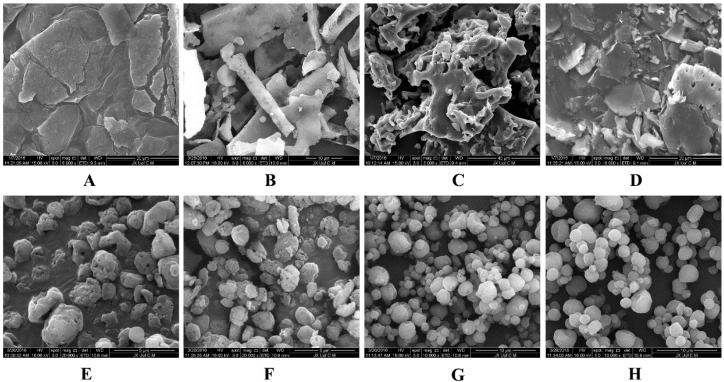
SEM morphology of the freeze-dried BCL-NC (**A**–**D**) and spray-dried BCL-NC (**E**–H) with different stabilizers/matrix formers: (**A**) 100% PNS; (**B**) 100% PVPK30; (**C**) 10% PNS + 400% Trehalose; (**D**) 10% PVPK30 + 400% Trehalose; (**E**) 100% PNS; (**F**) 100% PVPK30; (**G**) 10% PNS + 400% Lactose and (**H**) 10% PVPK30 + 400% Lactose.

Figure 6 shows the redispersibility of BCL-NS/10% PNS and BCL-NS/PVPK30 combined with different concentrations of cryoprotectants during lyophilization. The redispersibility of BCL-NC/100% PNS and BCL-NC/100% PVPK30 was 1.06 ± 0.15 and 1.02 ± 0.16, respectively. This indicated 100% concentration of PNS could prevent from irreversible aggregation of BCL-NS during lyophilization. Redispersibility of BCL-NC/10% PNS and BCL-NC/10% PVPK30 combined with 200% concentration of cryoprotectants (sucrose, trehalose and lactose) were very poor, but 10% of PNS combined with 400% concentration of sucrose and trehalose could improve the redispersibility of BCL-NC (RDI 1.17 ± 0.09) after lyophilization. Figure 7A–D shows the morphology of freeze-dried BCL-NC. It was seen that BCL-NC/100% PNS had a lamellar-shape structure (Figure 7A), but BCL-NC/100% PVPK30 appeared as rod-like aggregation (Figure 7B). The BCL-NC/10% PNS combined with 400% of trehalose possessed a porous aggregation (Figure 7C), but BCL-NC/10% PVPK30 combined with 400% of trehalose had a lamellar-shape structure (Figure 7D). Therefore, 10% of PNS combined with 400% concentration of trehalose can prevent irreversible aggregations of BCL-NC during freeze-drying.

### 2.3. Effect of PNS with or without Other Matrix Formers on Redispersibility of BCL-NC during Spray-Drying Process

**Figure 8:**
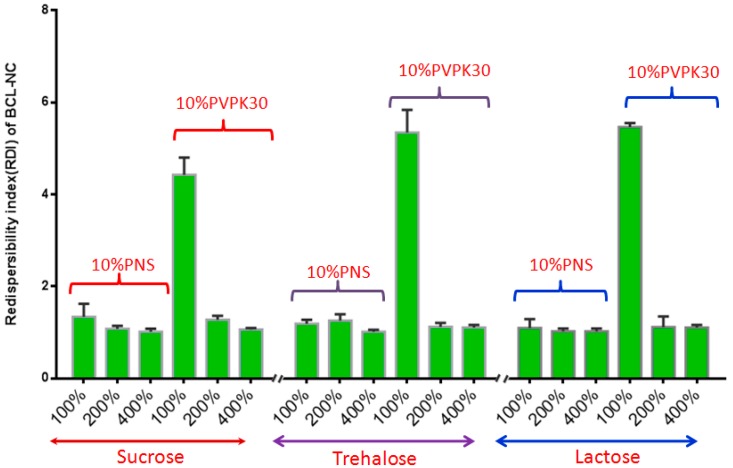
Redispersibility of BCL-NS/10% PNS and BCL-NS/10% PVPK30 combined with different concentrations of matrix formers during spray-drying (mean ± S.D., *n* = 3).

Spray-drying, a rapid solidification process for drug nanocrystals in which a feed solution containing the drug nanosuspensions is atomized into droplets that rapidly dry due to their high surface area and intimate contact with drying air. However, spray-drying is usually more aggressive compared with freeze-drying, so the effect of PNS on the redispersibility of BCL-NC was investigated. Figure 8 shows the redispersibility of BCL-NS/10% PNS and BCL-NS/10% PVPK30 combined with different concentrations of matrix formers (sucrose, trehalose and lactose) during spray-drying. The redispersibility of spray-dried BCL-NS/10% PNS with three matrix formers was smaller than those of BCL-NS/10% PVPK30, which indicated PNS possessed excellent performance for BCL-NS. Furthermore, high concentrations of matrix formers also played an important role on redispersibility of BCL-NS. The redispersibility of BCL-NS/10% PNS combined with 200% and 400% concentration of matrix formers were very satisfactory, compared with those with 100% concentration. Figure 7E–H shows the morphology of spray-dried BCL-NC. It was seen that spray-dried BCL-NC had a round-shape structure, but the rounding of BCL-NC/100% PNS and BCL-NC/100% PVPK30 particles were very poor (Figure 7E,F). BCL-NC/10% PNS and BCL-NC/10% PVPK30 combined with 200% lactose possessed an excellent roundness (Figure 7G,H). Meanwhile, 10% concentration of PNS combined with 200% lactose could improve the redispersibility of BCL-NC during spray-drying (Figure 8).

At present, PNS was used as an effective natural stabilizer for the BCL nanocrystals suspensions. It was observed that PNS was able to prevent the aggregation of the nanocrystals in the suspension state and irreversible agglomeration during freeze-drying/spray-drying process. This was the reason that PNS possessed the excellent interfacial property and electrostatic effect, which could adsorb onto the surface of BCL nanocrystals. PNS could be an effective nature stabilizer for production of drug nanocrystals. However, the in-depth mechanism behind the phenomenon is not yet well-understood in this study and systematically elucidated in the future. 

## 3. Experiment Section

### 3.1. Materials

Baicalein and Panax notoginseng saponins (consisting of approximately 50% ginsenoside Rb1, 19.5% ginsenoside Rg1, 9.0%ginsenoside Re and 8.5% notoginsenoside R1) were purchased from Zelang Co. (Nanjing, China). Polysorbate 80 (Tween 80, SHANHE, Anhui, China) and Hydroxypropylmethylcellulose (HPMC, Methocel E15LV PremiumEP^®^, Colorcon, Dartford, UK) were commercially obtained. Povidone 30 (PVPK30) was kindly donated by ISP (Toms River, NJ, USA). Sucrose, trehalose and lactose were obtained from DAMAO Chemical Co., Ltd. (Tianjin, China).

### 3.2. Production of BCL-NS

BCL-NS was prepared by high pressure homogenization. Before producing BCL-NS, BCL coarse powder (1%, *w/v*) was dispersed in a different dispersant solution of PNS, Tween 80, PVPK30 and HPMC (0.1%, *w/v*). The obtained mixture was disintegrated into coarse suspension by a high shear homogenizer (FLUKO^®^ FA25, Essen, Germany) at 19,000 rpm for 2 min. and the obtained coarse suspension was feed into a piston-gap high pressure homogenizer (AH-1000D, ATS Engineering Inc., Seeker, BVI, Canada), and continuously homogenized at 100 bar, 300 bar, 500 bar, 700 bar and 800 bar each for 30 cycles. Then the BCL-NS was obtained for further analysis.

### 3.3. Characterization of BCL-NS

#### 3.3.1. Particle Size Measurements

The determination of particles size was performed on a Mastersizer Micro Plus (Malvern Instruments Limited, Worcestershire, UK) that has a working range of 0.050–550 lm. Analysis of the diffraction patterns was done using the Mie model (“standard” presentation: dispersant refractive index = 1.33, real particle refractive index = 1.670, imaginary particle refractive index = 0.1). The average 10%, 50% and 90% volume percentile (D_10_, D_50_ and D_90_) were determined. Furthermore, the average span of the distribution [Span = (D_10_–D_90_)/D_50_] was calculated. All measurements were performed in triplicate.

#### 3.3.2. Zeta Potential Assay

Zeta potential was estimated using the Zetasizer nano-ZS (Malvern Instruments, Malvern, UK). The zeta potential was determined 3 times for each drug and averages and standard deviations were calculated.

#### 3.3.3. Stability Index (SI)

The stability of BCL-NS was evaluated by stability index (SI) as follows:

$$SI=\frac{D_{C}}{D_{0}}$$

where *D*_0_ is the particle size D_50_ value of the freshly prepared BCL-NS and *D_t_* is the corresponding D_50_ value of BCL-NS stored for one month at room temperature. D_50_ means that 50% of the particles are below the given size. An SI of near 1 usually means that BCL-NS is relatively stable.

#### 3.3.4. Determination of Surface Tension

Surface tension measurements of the stabilizer solutions were determined using the Wilhelmy plate method on a DCAT21 instrument (Dataphysics GmbH, Filderstadt, Germany). The experiment was performed in triplicate for each stabilizer solution measured.

### 3.4. Conversion BCL-NS into BCL-NC via Freeze-Drying

#### 3.4.1. Effect of PNS with or without Other Cryoprotectants on Redispersibility of BCL-NC during Freezing Process

The freeze stress conditions of BCL-NS stabilized by different stabilizers were shown in Table 3. The average particle sizes of frozen BCL-NC were determined at room temperature. Measurements were made in triplicate for all the measurement runs.

#### 3.4.2. Roles of PNS with or without Other Cryoprotectants on Redispersibility of BCL-NC during Lyophilization Process

The different concentrations (100%, 200% and 400%, relative to the weight of BCL) of cryoprotectants (sucrose, trehalose and lactose) were added into BCL-NC, which was stabilized by 10% concentration (relative to the weight of BCL) of PNS or PVPK30. BCL-NS were prepared according to Section 3.2 and frozen at −80 °C for 6 h. The BCL-NS were freeze-dried by means of freeze-dryer (FreezeZone^®^ Stoppering Tray Dryers, LABCONCO Corporation, Kansas, MO, USA). The applied cycle conditions were as follows: Freezing was performed at −40 °C (shelf inlet temperature) for 60 min. The shelf temperature ramp rates from the freezing step in to the primary drying step were 1 °C/min. Primary drying and secondary drying was performed at −20 °C and 10 °C (shelf inlet temperature) for 12 h, respectively. The shelf temperature ramp rates from the primary-drying step in to the secondary drying step were 1 °C/min.

#### 3.4.3. Conversion BCL-NS into BCL-NC via Spray-Drying

The BCL-NS were converted into BCL-NC by a Buchi mini spray dryer (model B290, Buchi Laboratoriums-Technik AG, Flawil, Switzerland). The spray-drying strength was set as follows: inlet temperature 125 °C, feed flow rate at 10 mL/min; aspiration rate at 75%; and atomizing air flow at 50 mmHg. The dried BCL-NC were separated from the drying air in the cyclone (57–83 outlet temperature) and deposited at the bottom of the collector. They were collected and kept at room temperature for future testing and evaluation.

### 3.5. Redispersibility (RDI) Index of BCL-NC

The dried BCL-NC powder (1 g) were dispersed in a 50 mL beaker containing 20 mL de-ionized water and gently agitated for 1 min. The particles size of nanosuspensions was measured according to Section 3.3.1. The redispersibility of BCL-NC was calculated as follows:

RDI=DD0

where *D*_0_ represents the volume-weighed mean particle size of the freshly prepared BCL-NS directly prior to solidification (freezing, lyophilization and spray-drying) and D represents the corresponding value of reconstituted BCL-NC post-solidification. An RDI of 1 would therefore mean that BCL-NC can be completely reconstituted into the original nanosuspensions after rehydration.

### 3.6. Scanning Electron Microscope (SEM) of BCL-NC

Morphological evaluation of representative BCL-NC was performed by means of scanning electron microscope (SEM) (Hitachi X650, Tokyo, Japan). All samples were examined on a brass stub using carbon double-sided tape. BCL-NC samples were glued and mounted on metal sample plates. The samples were gold coated (thickness ≈ 15–20 nm) with a sputter coater (Fison Instruments, Ipswich, UK) using an electrical potential of 2.0 kV at 25 mA for 10 min. An excitation voltage of 20 kV was used in the experiments.

## Figures and Tables

**Table 1:** Characterization of freshly prepared baicalein nanocrystals suspension (BCL-NS) stabilized by different stabilizers (mean ± S.D., *n* = 3).

Dispersants	BCL-NS/PNS	BCL-NS/PVPK30	BCL-NS/Tween 80	BCL-NS/HPMC
D_50_ (µm)	0.156 ± 0.012	0.145 ± 0.006	0.144 ± 0.004	0.149 ± 0.005
Span	3.145 ± 0.013	4.250 ± 0.011	4.264 ± 0.007	5.198 ± 0.012
SI	0.969 ± 0.012	1.021 ± 0.006	1.056 ± 0.004	1.443 ± 0.005
ZP (mV)	−40.1 ± 1.6	−31.7 ± 2.3	−33.4 ± 1.4	−29.1 ± 3.1

**Table 2:** Viscosity and surface tensions of the applied stabilizers (mean ± S.D., *n* = 3).

Stabilizer Concentrations (%, *w*/*w*)	Physicochemical Property					
	Viscosity (mPa s)			Surface Tension (mN/m)		
	50	25	10	50	25	10
PNS	5.02 ± 0.29	4.91 ± 0.17	4.49 ± 0.11	41.69 ± 0.32	41.92 ± 0.22	44.17 ± 0.28
PVPK30	6.10 ± 0.51	5.43 ± 0.37	5.01 ± 0.21	42.18 ± 0.56	45.06 ± 0.76	47.40 ± 0.84
Tween-80	5.08 ± 0.35	4.73 ± 0.29	4.61 ± 0.32	40.60 ± 0.55	40.71 ± 0.64	42.49 ± 0.71
HPMC	8.89 ± 0.32	8.23 ± 0.28	5.01 ± 0.41	42.15 ± 0.94	42.57 ± 1.02	43.04 ± 0.65

**Table 3:** The applied freezing stresses conditions for BCL-NS.

Temperature Strength	“Conservative”	“Moderate”	“Aggressive”
Freezing	−20 °C for 12 h	−80 °C for 6 h	−196 °C for 2 h
